# Anti-Inflammatory Activity of Bilberry (*Vaccinium myrtillus* L.)

**DOI:** 10.3390/cimb44100313

**Published:** 2022-09-30

**Authors:** Anshul Sharma, Hae-Jeung Lee

**Affiliations:** 1 Department of Food and Nutrition, College of Bionanotechnology, Gachon University, Seongnam-si 13120, Korea; 2 Institute for Aging and Clinical Nutrition Research, Gachon University, Seongnam-si 13120, Korea; 3 Department of Health Sciences and Technology, GAIHST, Gachon University, Incheon 21999, Korea

**Keywords:** bilberry, inflammation, anti-inflammatory, oxidative stress, anthocyanin

## Abstract

Inflammation is important in the pathogenesis of several chronic diseases. The anti-inflammatory properties of berries have been investigated but the anti-inflammatory activity of bilberry has received little attention and a detailed review is yet to be published. Therefore, we compiled information on the phytochemicals of bilberry and preclinical and clinical studies of its anti-inflammatory properties. The review was based on studies from 2007 to date. Phytoconstituents of bilberries were phenolic acids, organic acids, anthocyanins, coumarins, flavonols, flavanols, tannins, terpenoids, and volatile chemicals. Data from cell and animal model studies show that bilberry has an anti-inflammatory effect by lowering tumor necrosis factor-α, interleukin (IL)-6, and IL-1β expression, inducing nitric oxide synthases and cyclooxygenases, and altering the nuclear factor kappa B and Janus kinase-signal transducer and activator of transcription signaling pathways. Bilberry supplementation as fruits (frozen, processed, and whole), juices, and anthocyanins reduced levels of inflammatory markers in most clinical studies of metabolic disorders. Therefore, bilberry may be useful for the prevention and treatment of chronic inflammatory disorders.

## 1. Introduction

Inflammation is a medical term derived from the Latin phrase “inflammare”, meaning “to burn” [1]. Inflammation is important in the development and progression of noncommunicable conditions such as type 2 diabetes, cardiovascular disease (CVD), Alzheimer’s disease, and cancer. It is categorized as acute or chronic. Acute inflammation is of short duration and its key features are fluid exudation and leukocyte emigration, particularly of neutrophils. Chronic inflammation, by contrast, is of longer duration and is associated with lymphocytes, macrophages, fibrosis, and tissue necrosis [2]. Circulating immune cells subsequently target the area of the injured tissue to increase pro-inflammatory activity [3]. Pro-inflammatory mediators and cytokines such as nitric oxide (NO), interleukin-1β (IL-1β), prostaglandin E2 (PGE2), interleukin-6 (IL-6), and tumor necrosis factor-α (TNF-α) are typically responsible for a shift of the inflammatory response from acute to chronic, leading to chronic diseases. Thus, these cytokines and proinflammatory factors are targets for the discovery and evaluation of anti-inflammatory agents [1]. In the last two decades, research on the anti-inflammatory effects of bioactive compounds from fruits and vegetables has increased.

A series of coordinated, dynamic behaviors, including cellular and vascular processes, mediate inflammation [1]. The inflammatory response is triggered by changes in reactive oxygen species (ROS), bacterial endotoxins, viruses, fatty acids, carcinogens, and growth factors [4]. Innate immune cells recognize pathogens or inflammatory stimuli via specific transmembrane receptors known as pattern-recognition receptors (PRRs). PRRs recognize pathogen-associated molecular patterns (PAMPs, structures conserved in microbes) and damage-associated molecular patterns (DAMPs, endogenous molecules derived from internal injuries) [5]. Examples of PRRs include Toll-like receptors (TLRs), nucleotide-binding and oligomerization domain (NOD)-like receptors (NLRs), and C-type lectin receptors (CLRs) [5]. TLRs can detect lipopolysaccharide (LPS), a crucial component of the cell walls of Gram-negative bacteria linked to a number of inflammatory conditions. LPS stimulates the release of cytokines and other inflammatory mediators, leading to a proinflammatory state [6,7].

In response to LPS, nuclear factor-kappa B (NF-κB) translocates into the nucleus and induces the phosphorylation of mitogen-activated protein kinases (MAPKs). This leads to a response from the outside to the inside of the cell [8].

NF-κB complexes are attached to trimeric IκB kinase complex (IKK). IKK comprises two catalytic subunits (IκB kinase-alpha [IKKα] and IκB kinase-beta [IKKβ]) and a regulatory subunit termed IKKγ or NF-κB essential modulator (NEMO) [9]. IKK is stimulated by the binding of ligands such as cytokines, growth factors, damaged microbial cells, mitogens, or stress agents to cell-surface receptors. After activation, IKK phosphorylates IκBα (NF-κB inhibitory protein), leading to its ubiquitylation and proteosome-mediated degradation, activating NF-κB. Induction of NF-κB signaling is an important biochemical process that controls a series of events. Activated NF-κB translocates into the nucleus and stimulates the transcription of pro-inflammatory cytokines (acting as immune signaling molecules), including IL-1β, PGE2, IL-6, TNF-α, and NO, as well as inflammation-related enzymes such as cyclooxygenase-2 (COX-2) and inducible nitric oxide synthase (iNOS) [10,11].

The NF-κB transcription factor consisted of Rel protein family members including p65 (Rel-A), Rel-B, c-Rel, p50 (NF-κB1), and p52 (NF-κB2), which regulate the transcription of target genes [12,13]. The NF-κB p65 subunit is a target in inflammation because of the imbalanced surge in pro-inflammatory mediators [14]. The MAPK signaling pathway promotes NF-κB translocation (cytoplasm to the nuclei) and transactivation of proinflammatory mediators [15]. The MAPK family includes p38 kinase, extracellular signal-regulated kinase (ERK), and c-Jun N-terminal kinase (JNK). Therefore, this route is a potential anti-inflammatory therapeutic target [16]. Lifestyle factors such as poor diet, excess body fat, and excessive energy intake also contribute to inflammation [4].

Berries are commonly consumed fruits and contain a variety of nutritive and non-nutritive components, including polyphenols, vitamins, and minerals [17,18]. Phenolic compounds in berries, particularly anthocyanins, have anti-inflammatory, antioxidant, anti-diabetic, anti-cancer, cardio-protective, anti-Alzheimer, antimicrobial, and anti-obesity effects [18,19,20,21,22]. Several berry fruits are underutilized because they are harvested only in wild and local areas. Therefore, the health benefits of these underutilized berries need to be exploited. The anti-inflammatory effects of berries have been reviewed [4,20]. However, a targeted review highlighting the anti-inflammatory potential of bilberries is lacking. We review the anti-inflammatory properties of bilberry and its phytoconstituents, particularly anthocyanins. We first focus on bilberry, anthocyanins, bioactivity, and bioavailability based on animal and human studies. Next, we summarize the preclinical studies demonstrating bilberry’s anti-inflammatory potential. Finally, we summarize and discussed the therapeutic effects of anthocyanin-rich bilberry extract, focusing on clinical studies. We highlight the anti-inflammatory properties of bilberry and its extracts in health and in inflammation-related chronic conditions. Finally, we address effects on the proinflammatory indicators high-sensitivity C-reactive protein (hsCRP), adhesion molecules (vascular cell adhesion molecule 1 [VCAM-1] and intercellular adhesion molecule 1 [ICAM-1]), and cytokines and chemokines (IL-1β, TNF-α, IL-6, monokine induced by gamma interferon [MIG]).

The accumulated research shows how a treatment interacts with the system in both healthy people and patients. Such information is important to learn about dosages, side effects, and treatment safety. Healthy individuals are treated to get an early understanding about how to administer the medication to minimize risks and maximize potential benefits. Healthy people exhibit changes with a normal range of biomarker measurements and exhibit a somewhat flexible homeostasis. Several ways to measure the effects of nutritional constituents and bioactive compounds have been suggested. These include comparing changes in inflammatory markers to a normal range, preventing modulation caused by other factors (such as undernutrition), and changing from a less favorable range to a more favorable range [23]. Continuous study of both healthy individuals and patients will improve our understanding and pave the road for constructive research to identify solutions to chronic diseases.

## 2. Bilberry

**Figure 1:**
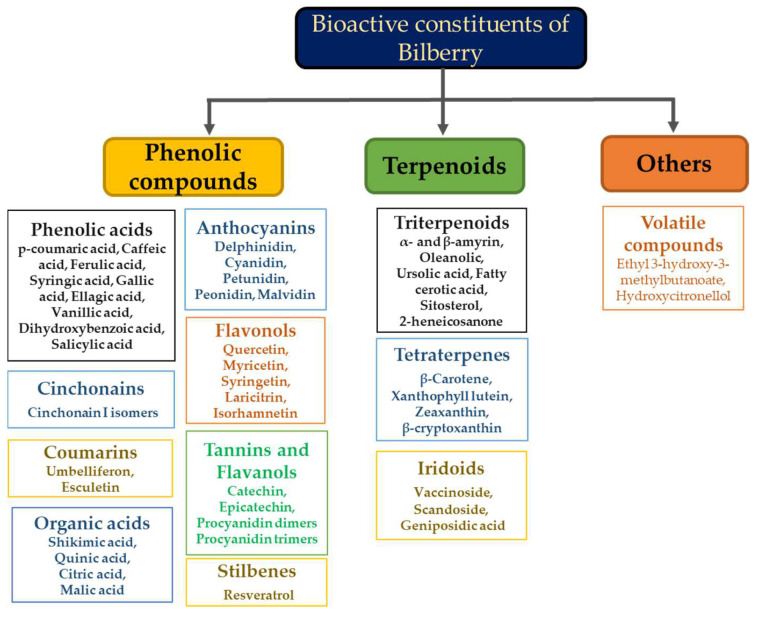
Bioactive constituents of bilberries [28].

Bilberry (*Vaccinium myrtillus* L.) is a dwarf shrub of the genus *Vaccinium* that is also known as huckleberry, whortleberry, and European blueberry. However, older texts and the European Pharmacopoeia refer to bilberry as *Myrtilli fructus* [24]. The Danish word bollebar, which denotes a dark fruit, is the first appearance of the name bilberry [25]. Since ancient times, bilberries have been used to cure skin ulcers, hemorrhoids, nausea, vomiting, diarrhea, and inflammation of the mucosal tissues. They are also believed to improve eyesight [24]. Bilberry is a perennial shrub of the Ericaceae family that is native to northern Europe and North America, but has also been reported in Asia [26]. The shrub can reach a height of 35 to 60 cm. The blue/black fruit is spheroidal and about 5–9 mm in diameter [27,28]. It is challenging to grow bilberries because of their poor environmental tolerance. Furthermore, shrub growth and berry yield is affected by numerous environmental elements, such as meteorological conditions, soil type, light, or cultivation conditions [29,30]. The berries contain numerous tiny, shiny, brownish-red seeds [25]. Bilberries reportedly contain sugars, vitamins, pectin, and phenolics. The phenolic compounds include anthocyanins (biggest fraction), terpenoids (triterpenoids, tetraterpenes, and iridoids), flavonols (quercetin and myricetin), tannins and flavanols (catechin and epicatechin), coumarins, phenolic acids, and resveratrol (Figure 1) [31]. The phenolic content of bilberry fruits from northern latitudes is higher than those from southern latitudes [32]. The most abundant anthocyanins in bilberry include delphinidin and cyanidin, followed by petunidin, peonidin, and malvidin [31]. Bilberry contains few anthocyanidins, almost all as glycosides [33].

Bilberry is available as fresh, whole dried, and frozen berries, as well as industrial products, including preserves, juices, jams, and powdered or liquid concentrates, which are increasingly used as food supplements [27]. Bilberry is commonly known as a “super food” or “functional food,” due to the abundance of its health-promoting compounds [31]. The phytoconstituents of bilberry have antioxidant, anticancer, anti-obesity, anti-inflammatory, anti-diabetic, antimicrobial, eye-protective, cardioprotective, and neuroprotective activities [31,34,35,36,37,38]. The bioavailability and distribution of bilberry compounds have been investigated in animal models and humans. In one study, rats administered an anthocyanin-rich bilberry extract (1.43 mmol/kg body weight) had considerably higher plasma antioxidant activity than control rats [39]. Together with unidentified metabolites, anthocyanins in their intact glycosidic forms were found in urine. Bilberry anthocyanins and their metabolites were excreted in urine at a rate of 0.71 μmol/24 h. Cecal contents contained bilberry anthocyanins and their associated aglycones. Consumption of bilberry extract increased plasma antioxidant activity despite its modest bioavailability [39].

According to Sakakibari et al. [40], 15 min after the consumption of bilberry extract (0.5%) for 2 weeks, the plasma levels of these anthocyanins in male mice peaked at 0.26 µmol/L, followed by a marked decline. Two primary anthocyanins, malvidin-3-galactoside and malvidin-3-glucoside, were detected in plasma. Anthocyanins were detected in the liver, lung, kidney, and testes, but not in the brain, eyes, heart, muscle, and fat, suggesting the organotropic behavior of bilberry anthocyanins. The same group conducted two experiments on human volunteers with and without a colon [41]. In one of those experiments, anthocyanins from an oral dose of bilberry extract (4.95 mmol anthocyanins) were swiftly absorbed, and their bioavailability after 8 h of consumption was relatively low (0.02% plasma). During a 24 h period, 0.03 ± 0.02% of the ingested anthocyanins were detected in urine. Anthocyanins entered the bloodstream primarily as glucuronides, particularly of the peonidin (46%) and malvidin (28%) types. Despite higher concentrations in the first extract, petunidin, delphinidin, and cyanidin were detected in trace amounts in plasma. This could have been a result of methylation and dihydroxylation [42]. The levels of several degradants are approximately 20- and 38-fold greater in plasma and urine, respectively, relative to parent anthocyanins [41]. The major degradation products are vanillic acid and synergistic acids (from peonidin and malvidin) [43]. The levels of anthocyanins and other products in the plasma and urine of patients with an intact stomach are considerably higher than in those with ileostomies [41]. In one study [44], volunteers were treated with bilberry extract encapsulated with whey protein or citrus pectin. Encapsulation had no discernible effect on the bioavailability of bilberry anthocyanins, but there were modulatory effects. Whey protein encapsulation modulated short-term bioavailability and citrus pectin encapsulation enhanced bioavailability during transit through the small intestine and altered the level of the breakdown product phloroglucinol aldehyde in human plasma. Urine contained more anthocyanins (108%) and serum contained less (28%) anthocyanins relative to nonencapsulated extract. However, the study had a small sample size and inconsistent data on newly found metabolites and breakdown products.

## 3. Search Strategy

A literature search was executed using PubMed, Google Scholar, and the Science Direct depositories for relevant information between 2007 and August 2022. The following keywords were used to identify relevant in vitro, in vivo, and clinical studies on bilberry and its anti-inflammatory activity: “bilberry” or “*Vaccinium myrtillus*” or “bilberry and inflammation” or “bilberry and clinical study” or “bilberry and metabolic syndrome” or “bilberry and cardiovascular diseases,” or “*Vaccinium myrtillus* and inflammation” or “bilberry and chronic disease,” or “bilberry and ulcerative colitis” or “hypercholesterolemia.” The literature search yielded 13 preclinical and 11 clinical studies up to August 2022. A single in vivo study evaluated grape and bilberry juice, and two clinical studies assessed anthocyanins (bilberry and blackcurrant) and bilberry/red grape preparation.

## 4. Anti-Inflammatory Activity

Anti-inflammatory activity is mediated by the targeting of inflammatory factors or pro-inflammatory cytokines [1]. Natural dietary factors have a variety of positive health effects, particularly in the mitigation of inflammatory, pathogenic, and chronic conditions [45]. Berries, among the most popular fruits in the human diet, contain natural substances such as anthocyanins, flavonoids, vitamins, dietary fibers, micronutrients, and volatile organic compounds, which have anti-inflammatory effects in cell, animal, and human models [4,20]. In vitro and in vivo studies of bilberry’s anti-inflammatory activity are discussed below.

### 4.1. In Vitro and In Vivo Studies

Preclinical research indicates that bilberries can reduce inflammation, an effect possibly mediated by its phytoconstituents, particularly anthocyanins. The anti-inflammatory effects of bilberry and its extracts in cell and animal models are listed in Table 1.

Analysis of the effects and mechanisms of naturally occurring bioactive substances at the genome level has been much enhanced by DNA microarray technology. Chen et al. [46] used a bilberry extract to attenuate the gene expression profile of LPS-induced RAW264.7 cells (inflammatory cell model). An oligonucleotide DNA microarray was used to examine inflammation- and cell defense-related genes. The results suggested that anthocyanins target proinflammatory genes, thereby inducing an anti-inflammatory effect.

Oxidative stress and inflammation are closely related, and both contribute to cell death, which is linked to several chronic diseases [47,48,49]. Phytoconstituents of medicinal plants have anti-inflammatory effects in chronic diseases [50]. Pomari et al. [51] assessed the effect of bilberry extract on the viability of RAW264.7 cells using escalating dosages of hydrogen peroxide (H_2_O_2_) and extract with various incubation durations. H_2_O_2_ at 200 μM was used based on its induction of the expression of COX2, IL-1β, nuclear factor erythroid-derived 2-like 2 (NFE2L2), NF-κB1, NF-κB2, nitric oxide synthase 2 (NOS2), and TNF-α and suppression of peroxisome proliferator-activated receptor γ expression. The extract downregulated COX2, IL-1β, NF-κB1, NF-κB2, TNF-α, and NOS2 expression and upregulated NFE2L2 [51] (Table 1). A study of the anti-inflammatory and antioxidant effects of hydroethanolic extracts of bilberry on LPS-stimulated RAW 264.7 cells showed that six anthocyanins downregulated iNOS, COX-2, TNF-α, and IL-6 and suppressed NO generation [52]. The extract at 2 mg/mL inhibited lipid peroxidation by 89% at 88 h compared to 97.7% for ascorbic acid (2 mg/mL). It also showed radical-scavenging activity (Table 1).

Polyphenols attenuate disease activity in inflammatory bowel disease (IBD) [53]. Roth et al. [54] demonstrated that bilberry extract reduced interferon gamma (IFN-γ)-induced signal transducer and activator of transcription (STAT)1 and STAT3 activation as well as proinflammatory cytokine gene expression in human monocytes (THP-1). IFN-γ triggers intracellular signaling pathways such as the Janus kinase (JAK)-STAT pathway [55]. Therapeutic efficacy in IBD is linked to the blocking of such signaling pathways. In IFN-γ-induced THP-1 cells, bilberry anthocyanins inhibit the activation of this pathway. Furthermore, TNF-α activates ERK in THP-1 cells. By contrast, bilberry extract does not affect TNF-α-induced cytokine expression. Nardi et al. [56] assessed TNF-α-induced phosphorylation of NF-κB enhanced by bilberry in mice (Table 1) and found that extract at 50 and 200 mg/kg reduced paw edema by 28.8%. Injection of carrageenan induced neutrophil translocation to the inflamed paw and the secretion of enzymes such as myeloperoxidase (MPO). Such inflammatory-related processes cause neutrophil buildup in affected tissues, which can intensify the inflammatory reaction. The bilberry preparation modulated inflammation by reducing MPO production and the byproducts of lipid peroxidation. In addition, the extract ameliorated acute inflammation by preventing ROS generation [56]. Among 99 ethanolic plant extracts, Schink et al. [57] found that *V*. *myrtillus* had a marked anti-inflammatory effect and enhanced the viability of HeLa-TLR4, THP-1, and human embryonic kidney (HEK)TLR2/HEK-TLR4 cell lines [57]. Luo et al. [58] reported that anthocyanin-rich bilberry extracts had anti-inflammatory effects on liver inflammation in mice, leading to suppression of LPS-induced iNOS, TNF-α, IL-1β, and IL-6 transcript levels and iNOS, TNF-α, and NF-κB protein levels [58] (Table 1).

Uveitis (Latin: ūva) is the inflammation of intraocular tissue, which is composed of the iris, ciliary body, and choroid. It is categorized as infectious and noninfectious [59]. Treatment with an endotoxin such as LPS induces uveitis in animal models, known as endotoxin-induced uveitis (EIU). Yao et al. [60] showed that bilberry extract protected against LPS-induced retinal inflammation. Mice fed bilberry for 5 days showed dose-dependent decreases in NO and malondialdehyde levels, and increased expression and activity of antioxidant factors [60]. Therefore, anthocyanins in bilberries can reduce LPS-induced oxidative stress in EIU.

A protective effect of bilberry extract and its anthocyanin fraction has been demonstrated in Balb/c mice with experimental colitis (acute and chronic). Administration of bilberries had encouraging effects on numerous factors, particularly in acute colitis induced by dextrane sodium sulphate (DSS). Bilberries contain 10% anthocyanins, which have antioxidant, anti-inflammatory, and anti-carcinogenic effects, and can ameliorate acute and chronic colitis. The authors advocated evaluation of the clinical efficacy of the studied components in IBD patients [61]. Triebel et al. [62] evaluated the effects of bilberry extract, anthocyanins, and anthocyanidins on human colon epithelial cells (T 84) stimulated with TNF-α, IFN-γ, and IL-1β. Bilberry extract and single anthocyanins considerably decreased the production of pro-inflammatory mediators linked to IBD. In addition, cyanidin-3-glucosides, as opposed to anthocyanidins, were stable in cell culture [62].

Low-grade inflammation and hypertension are linked to multiple comorbidities, including obesity. Low-grade chronic inflammation is related to a high-fat diet (HFD), which is mediated by the interactions of multiple cell types and mediators, including cytokines and adipokines [63]. A study tested the effects of different concentrations of freeze-dried bilberries on HFD-induced development of obesity and inflammation. Treatment with bilberry extract amended the pro-inflammatory response induced by an HFD by decreasing IFNγ-producing T-cells [64]. Chemokines and neutrophils are essential in the inflammatory cascade. In one study, mice were fed an HFD for 24 weeks and subsequently administered an anthocyanin-rich bilberry meal (0.1% *w*/*w* anthocyanins); at 4 weeks, TNF-α expression in adipose tissue was decreased and the plasma level of neutrophil chemoattractant keratinocyte-derived chemokine (KC, also known as C-X-C motif chemokine ligand 1 [CXCL1]) was increased [65]. These effects explained the tissue-specific and transient anti-inflammatory activities, respectively. However, there was no discernible benefit in terms of adiposity or body weight reduction. In another study, feeding Fischer rats anthocyanin-rich grape bilberry juice for 10 weeks reduced the serum levels of triglycerides, cholesterol, resistin, and leptin, but had no effect on the release of adiponectin or other adipokines from adipose tissue [66].

**Table 1:** Anti-inflammatory effects of bilberry extracts in cell and animal models.

Ref.	Treatment	Model	Effects
[46]	Pretreatment with bilberry (40% anthocyanins), 75 µg/mL, 30 min + 40 ng/mL LPS for 6 h	RAW264.7	↓TNF-α, ↓IL-1β, ↓IL-6, ↓TNC, ↓PTGS2, ↓COX-2, ↓CCL22, ↓IFI47, and ↓IFI1
[51]	H_2_O_2_ (200 μM) + bilberry extract (1, 10, 100 and 200 μg/mL), 24 h	RAW264.7	↓COX2, ↓IL-1β, ↑NFE2L2, ↓NF-κB1, ↓NF-κB2, ↓TNFα, inhibited NOS2
[52]	LPS (1 μg/mL) with hydroethanolic extracts (400, 800 μg/mL), 24 h	RAW 264.7	No cytotoxicity, ↓NO, ↓COX-2, ↓iNOS, ↓IL-6 and ↓TNF-α. IC_50_ (μg/mL) = DPPH (151.98 ± 1.41), ABTS (57.15 ± 0.95)
[54]	Bilberry extract 20 min prior to TNF-α or IFN-γ (100 ng/mL each)	THP-1	↓p-STAT1, ↓p-STAT3, ↓MCP-1, ↓IL-6, ↓TNF-α, ↓ICAM-1, ↓T-bet (transcription factor)
[56]	Carrageenan (450 μg/paw) 50 and 200 mg/kg, 10 days	Mice	Paw edema reduction at low dose = 35.4% and high dose = 28.8%
[57]	Extract concentration 0.01% to 3%, 2 h followed by LPS (25 ng/mL), 8 h HeLa-TLR4, THP-1 (50 ng/mL, 4 h), HEKTLR2/HEK-TLR4 (100 ng/mL overnight incubation)	THP-1, HeLa-TLR4, HEKTLR2/HEK-TLR4	↑ cell viability, anti-inflammatory effects
[58]	LPS (0.5 mg/kg)-induced liver damage. 50, 100 and 200 mg/kg/day, 7 days	Kunming mice	↓plasma ALT and AST, ↓histopathogical injury, ↓TNF-α, ↓IL-1β, ↓IL-6, ↓iNOS, ↓NO, ↓NF-κB1, ↓ MDA in the liver
[60]	Pretreatment of bilberry−50, 100, 200 mg/kg/day, 5 days + LPS (100 mg/mouse), 24 h	BALB/C mice	↑ORAC, ↑GSH, ↑vitamin C, ↑SOD ↑GPx, ↓MDA
[61]	Acute colitis (2.5% DSS, 7 days), chronic colitis (2.5% DSS, 4 cycles for 7 days interrupted by 7 days). Feed + 20% dried bilberries (10% AC extract)	BALB/C mice	Preserved normal colon length ↓IFN-γ, ↓TNF-α, ↓IL−6
[62]	IFN-γ (10 ng/mL), IL-1β (5 ng/mL), TNF-α (10 ng/mL), 4 or 16 h. Anthocyanins (25−300 μM, 4 h) or BE (10−500 μg/mL, 24 h)	T84	↓TNF-α, ↓IP-10, ↓I-TAC, ↓sICAM-1, ↓GRO-α
[64]	HFD + 5% or 10% (*w*/*w*) of whole bilberries (BB), 24 weeks	C57BL/6J mice	↓Weight gain, ↓Th1 cells
[65]	HF diet. Bilberry anthocyanins dose: 0.1% *w*/*w* (35% ACY), 24 weeks	C57BL/6J mice	↓TNF-α
[66]	Anthocyanin rich grape and bilberry juice, 1551 mg ACY/L (15 mg ACY per day), 10 weeks	Fischer rats	↓serum cholesterol, ↓TG, ↓leptin, ↓resistin No changes in adiponectin, secretion of adipokines from adipose tissue

ABTS: 2, 2′-azino-bis (3-ethylbenzothiazoline-6-sulfonic acid); ALT: alanine aminotransferase; AST: aspartate transaminase; CCL22, CC chemokine ligand 22; COX-2: cyclooxygenase-2; DPPH: 2,2-Diphenyl-1-picrylhydrazyl; DSS: dextrane sodium sulphate; GPx: glutathione peroxidase; GRO-α: growth-related oncogene-alpha; GSH: glutathione; H_2_O_2_: hydrogen peroxide; HFD: high-fat diet; I-TAC: interferon-inducible T cell alpha chemoattractant; IC_50_: half-maximal inhibitory concentration; IFI, interferon-inducible protein; IL: interleukin; iNOS: inducible nitric oxide synthase; IP-10: interferon gamma-induced protein 10; LPS: lipopolysaccharide; MCP-1: monocyte chemoattractant protein-1; MDA: malondialdehyde; NF-κB: nuclear factor kappa-light-chain-enhancer of activated B cells; NFE2L2: NFE2 Like BZIP transcription factor 2; NO: nitric oxide; NOS2: nitric oxide synthase 2; ORAC: oxygen radical absorbance capacity; p-STAT: phosphorylated signal transducer and activator of transcription; PTGS2: prostaglandin-endoperoxide synthase 2; sICAM-1: soluble intercellular adhesion molecule-1; SOD: superoxide dismutase; TG: triglyceride; TLR: Toll-like receptor; TNC: tenascin; TNF-α: tumor necrosis factor-alpha. ↑: increased; ↓: decreased.

### 4.2. Clinical Studies

Bilberry fruit, juice, and powder have shown anti-inflammatory and antioxidant effects in clinical studies (Table 2). Karlsen et al. [67] evaluated the effect of bilberry juice consumption (330 mL per day for 4 weeks) in adults (*n* = 62) at high risk for CVD [67]. Compared to water controls, bilberry supplementation reduced the levels of NF-κB targets such as circulating hsCRP, IL-6, and MIG at 4 weeks, which were likely positively correlated. In addition, there was an unexpected increase in the plasma level of TNF-α. However, there were no effects on clinical factors, antioxidant status, or free radical stress. The bilberry group had higher plasma levels of quercetin and p-coumaric acid. In addition, the polyphenols quercetin, resveratrol, and epicatechin (1, 10, 25, and 50 mol/L, 30 min) suppressed NF-κB activation in human monocytic cells treated with LPS (1 μg/mL, 6 h) (Table 2) [67]. Lehtonen et al. [68] reported that consumption of 100 g (frozen) whole bilberries (corresponding to 100 g fresh fruit) for 33–35 days reduced the plasma levels of TNF-α in overweight and obese women (*n* = 110) [68]. In addition, bilberries decreased the plasma levels of VCAM-I and adiponectin but did not affect those of hs-CRP, IL-6, and soluble ICAM-1. Levels of circulating adiponectin (adipocyte-secreted cytokines) are positively correlated with insulin sensitivity and inversely correlated with body fat and inflammation [69]. Therefore, adiponectin is negatively correlated with the risk for CVD. The lower adiponectin level following bilberry ingestion may not have been caused by bilberry but by an inadequate interval from ingestion to assessment of circulating adiponectin levels [68] (Table 2).

Inflammation, dyslipidemia, obesity, and hyperglycemia are risk factors for metabolic syndrome (MetS), a global health issue [70]. Few human studies have evaluated the effect of bilberry on MetS. However, in one study, the addition of fresh bilberry (400 g daily, equivalent to 200 g berry puree and 40 g dried berries) to the diets of men and women with MetS significantly reduced the serum levels of hsCRP and IL-12 and showed a tendency to reduce those of IL-6 and LPS compared to the controls (regular diet with periodic consumption of ≤80 g fresh berries daily) [71]. Leptin and adiponectin levels were not affected by bilberry supplementation (Table 2). Therefore, the low-grade anti-inflammatory properties of bilberry may ameliorate MetS.

Aboonabi et al. [72] reported that consumption of 320 mg pure anthocyanins (MEDOX^®^ food supplement capsules, ~100 g fresh bilberries) daily for 4 weeks significantly reduced inflammation in subjects with the MetS and improved their lipid profile, as indicated by lower levels of inflammatory biomarkers, fasting blood glucose, low-density lipoprotein (LDL)-cholesterol, triglycerides, and serum levels of cholesterol. In addition, there was a significant reduction of the hsCRP level in females [72]. Another intervention study discovered these positive effects by dropping inflammation and ameliorating lipid and glucose metabolism [73]. These positive effects are likely a result of anthocyanin-mediated control of the production, activation, and activity of pro-inflammatory mediators, including NF-κB signaling pathways, pro-inflammatory cytokines (such as IL-6, IL-1α, and TNF-α), and pro-inflammatory enzymes such as COX-2. Reduced hsCRP synthesis was also noted. Anthocyanins are implicated in the nuclear factor erythroid 2-related factor 2 (Nrf2) and peroxisome proliferator-activated receptor (PPARs) pathways. Anthocyanins increased the expression of superoxide dismutase (SOD) and peroxisome proliferator-activated receptor-gamma (PPAR-γ). By regulating dietary fat and glucose metabolism, adipocyte differentiation, and inflammatory responses, PPAR-γ maintains healthy levels of lipid and glucose [73].

The anti-inflammatory effects of bilberry anthocyanins may facilitate recovery from exercise. A study provided evidence of the effects of bilberry juice on exercise-induced muscle damage (EIMD) and inflammation in recreational runners targeted for a half marathon [74]. However, intake of bilberry juice temporarily exacerbated muscle soreness and inflammation. Larger studies are needed to confirm these findings.

Anthocyanin supplements improve the lipid profile by increasing the level of high-density lipoprotein cholesterol (HDL-C) and decreasing that of LDL-cholesterol when taken for 8 and 12 weeks, respectively [75,76]. Zhu et al. [77] showed that 24 weeks of dosing with a processed anthocyanin mixture (320 mg/day bilberry and blackcurrant) decreased the serum levels of IL-1β, hsCRP, sVCAM-1, and LDL-C and increased that of HDL-C in hypercholesterolemic subjects, suggesting an anti-inflammatory response. Purified anthocyanin mixtures of delphinidin-3-O-β-glucoside (D3G) and cyanidin-3-O-β-glucoside (C3G) decreased IL-6 (20 ng/mL) and IL-1β (10 ng/mL)-stimulated CRP production and LPS-stimulated VCAM-1 secretion in HepG2 (human hepatoma) cells in a dose-dependent manner. The mixture had a greater effect than the individual anthocyanins and all three dose-dependently reduced LPS-stimulated VCAM-1 expressions in porcine iliac artery endothelial cells. This suggests that anthocyanins have additive anti-inflammatory effects. The effect of long-term treatment with pure anthocyanins on inflammation in hypercholesterolemic subjects was first demonstrated in that study.

Chronic inflammatory disorders could be ameliorated by reducing NF-κB activation and subsequently the inflammatory response. Accordingly, treatment with anthocyanins from bilberry and black currants (Medox, 300 mg/day [mixture of 3-O-rutinosides of delphinidin, and cyanidin and 3-O-β-glucosides, 3-O-β-galactosides, and 3-O-β-arabinosides of delphinidin, cyanidin, petunidin, peonidin, and malvidin, equivalent to ~100 g fresh bilberries daily]) for 3 weeks reduced NF-κB transactivation and the levels of pro-inflammatory mediators in 120 healthy male and female volunteers. In addition, co-administration of Medox and LPS to human monocytes reduced NF-kB activation by 27.6% and p65 DNA binding by 17.8% [78]. Therefore, bilberry anthocyanins have anti-inflammatory effects by modulating NF-κB activation.

Ulcerative colitis results in an overabundance of neutrophils and macrophages, leading to ROS production in inflamed colons. In an open-pilot trial, an anthocyanin-rich bilberry preparation had a significant anti-inflammatory effect by reducing mucosal inflammation, significantly decreasing the fecal calprotectin level, and modulating inflammatory changes in UC [79]. In a continuation study [80], IFN-γ induced the expression of IFN-γ receptor 2 (R2) in human THP-1 monocytes in vitro and reduced the expression of IFN-γ R2 in the UC intestine. In another study, colon biopsies and serum samples from a subject of a prior study [79] were evaluated for T-cell-derived cytokine expression [80]. Anthocyanin treatment reduced IFN-γ, TNF-α, and IFN-γ R2 expression, and the serum monocyte chemoattractant protein-1 (MCP-1) level, and enhanced IL-10 expression [80].

As free radical stress and inflammation are closely related, an inflammatory response increases ROS production, maintaining a pro-inflammatory environment. In one study, bilberry/red grape juice consumption (660 mL per day) improved oxidative stress and inflammation biomarkers in a double-blind, placebo-controlled, intervention study (*n* = 60) [81]. The intervention decreased the levels of biomarkers of inflammation (epidermal growth factor [EGF], IL-6, TNF-α, macrophage inflammatory protein [Mip]1β, and vascular endothelial growth factor [VEGF]) and tissue damage (lactate dehydrogenase [LDH]); however, the memory score was unaffected. In addition, the plasma levels of polyphenols (p-coumaric acid, hippuric acid, 3-hydroxypropionic acid, vanillic acid, and protocatechuic acid) were increased in the treatment compared to the placebo group. This suggested preventive effects on chronic inflammation and oxidative stress. The authors suggested that longer-duration studies were needed (Table 2).

**Table 2:** Effects of bilberry on biomarkers in humans.

Ref.	Design and Duration	Number	Subjects Type	Age (Y)	Dose/Day and Format	Biomarkers
[67]	Parallel, 2 –arm, controlled, 4 weeks	62	Males and females. CVD at risk	30–70	300 mL juice per day	↓IL-6, ↓IL-15, ↓ hsCRP, ↓MIG, ↑ TNF-α (unexpected)
[68]	Crossover, 2 phase, baseline controlled, 33 to 35 days	110	Females, overweight and obese	44.2	~100 g of frozen and whole	↓ TNF-α, ↓ sVCAM-1, ↓ adiponectin
[71]	Parallel, 2 –arm, controlled. 4 weeks run-in period followed by 4-week recovery period	27	Males and females with MetS features	53	60 g of freeze-dried powder, water slurry	↓ hsCRP, ↓ IL-12, ↓ IL-6, ↓ circulating LPS
[72]	Randomized controlled trial, 28 days	55	Normal healthy and MetS	25–75	MEDOX^®^ food supplement capsules, 320 mg per day	↓FBG, ↓TG, ↓LDL-C, ↓hsCRP, ↓ ADP-induced platelet
[73]	Clinical trial, 4 weeks	35	Normal and MetS	25–75	MEDOX^®^ (purified anthocyanins) 320 mg per day	↓ hsCRP, ↓TNF-α, ↓IL-6, ↓IL-1 α, ↓ COX-2, ↓ FBG, ↓TC, ↓TG, ↓LDL-C, ↑ PPAR-γ
[74]	Single blind, randomized, placebo-controlled, parallel study, 8 days	21	Males and females, recreational runners	--	200 mL juice twice per day	↑ moderate CRP 24 h post-race (unexpected)
[77]	Randomized placebo controlled, double-blinded trial, 24 weeks	150	Hypercholesterolemia subjects	40–65	Anthocyanins (320 mg/day) purified from bilberry and blackcurrant	↓ hsCRP, ↓ sVCAM-1, ↓ IL-1β, ↓ LDL-C, ↑ HDL-C
[78]	Randomized controlled trial, 3 weeks	120	Aged healthy volunteer men and women	40–74	2 Medox capsules of 75 mg 2 times per day	↓IL-8, ↓ RANTES, ↓IFN-α, ↓ IL-4, ↓IL-13
[79]	Open pilot trial, 6 weeks	13	Patients with mild to moderate UC	18–65	160 g of bilberry preparation	↓Fecal calprotectin levels, ↓endoscopic Mayo score
[80]	Open pilot trial *, 6 weeks	13	Patients with mild to moderate UC	18–65	160 g of bilberry preparation	↓IFN-γ, ↓TNF-α, ↓p-p65-NF-κB, ↑IL-22, ↑IL-10
[81]	Double blind, placebo-controlled intervention study, 9 weeks	60	Aged men with SMI	≥ 67	330 mL of bilberry/red grape preparation, 2 times a day	↓IL6, ↓TNF-α, ↓EGF, ↓Mip1β, ↓VEGF

ADP: adenosine diphosphate; CK: creatinine kinase; COX-2: cyclooxygenase-2; CVD: coronary artery disease; EGF: epidermal growth factor; FBG: fasting blood glucose; HDL-C: high-density lipoprotein cholesterol; hsCRP: high-sensitivity C-reactive protein; IFN-γ: interferon gamma; IL-1α: interleukin 1 alpha; IL: interleukin; LDL-C: low-density lipoprotein cholesterol; LPS: lipopolysaccharide; MetS: metabolic syndrome; MIG: monokine induced by interferon gamma; Mip1β: macrophage inflammatory protein-1 beta; NF-κB: nuclear factor kappa-light-chain-enhancer of activated B cells; PECAM-1: platelet endothelial cell adhesion molecule-1; PPAR-γ: peroxisome proliferator- activated receptor gamma; RANTES: regulated on activation, normal T-cell expressed and secreted; SMI: subjective memory impairment; SOD: superoxide dismutase; sVCAM-1: soluble vascular cell adhesion molecule-1; TC: total cholesterol; TG: triglyceride; TNF-α: tumor necrosis factor-alpha; UC: ulcerative colitis; VEGF: vascular endothelial growth factor. * Intestinal tissue specimens were obtained during the open-label bilberry ingestion pilot study and stirred at −80°. ↑: increased; ↓: decreased.

Most human studies have shown bilberry to have anti-inflammatory activity; however, these works have had some limitations. There has been significant heterogeneity, possibly caused by the use of different regimens, doses, durations, centers, and populations. Many studies have had significant bias, which should also be taken into consideration. Furthermore, the small number of studies that have been published means that the supporting evidence is sparse.

Bilberry fruit is classified as a Class 1 herb in the American Herbal Products Association’s (AHPA) Botanical Safety Handbook, which means that it can be ingested without risk if used as directed [82]. Bilberry and bilberry extracts are not associated with adverse effects [67,79]. Larger studies are needed to evaluate the therapeutic potential of bilberry in inflammation and related diseases.

## 5. Conclusions and Future Directions

We reviewed the anti-inflammatory effects of bilberry and its extracts based on preclinical and clinical studies. Bilberry reduces inflammation by downregulating the expression of pro-inflammatory factors (TNF-α, IL-1β, and IL-6) and enzymes (iNOS, COX-2), modulating signaling pathways, and reducing ROS levels in cell and animal models. In humans, bilberry in the form of juices, fresh/frozen berries, freeze-dried preparations, and anthocyanin extract (MEDOX^®^) ameliorate inflammation. Most studies have shown that anthocyanins reduce inflammation induced by LPS, TNF-α, and IFN-γ. Furthermore, anti-inflammatory effects have been observed in both healthy volunteers and patients with inflammation-associated chronic diseases. However, human studies have been small and of short duration. Therefore, human trials of bilberry extracts with a large population, of long duration, and with appropriate follow-up are needed. To prevent the loss of therapeutic effects, the processing and physical characteristics of bilberry need to be optimized. A rise in particular microorganisms such as *Desulfovibrio* and *Akkermansia*, following natural compounds intake, are believed to possess anti-inflammatory properties [83]. Therefore, studies of the effect of bilberry on the gut microbiota are needed. In conclusion, bilberry improves the levels of markers of inflammation and oxidative stress in humans.

## Data Availability

Not applicable.
